# Dr. Theodore Woodward Barton

**Published:** 1908-01

**Authors:** 


Dr. Theodore Woodward Barton, of Waterford, Pa., died at his home November 15, 1907, of pneumonia, aged 71 years. He graduated at the University of Buffalo in 1862; was a member of the Medical Society of the State of Pennsylvania and of the Medical Society of the County of Erie in that state.

